# Comparative gastrointestinal adverse effects of GLP-1 receptor agonists and multi-target analogs in type 2 diabetes: a Bayesian network meta-analysis

**DOI:** 10.3389/fphar.2025.1613610

**Published:** 2025-09-19

**Authors:** Xingmiao Xie, Shuiyuan Yang, Shuzhen Deng, Yuying Liu, Zhibin Xu, Binghong He

**Affiliations:** ^1^ Department of Pharmacy, Guangzhou Panyu District Eighth People’s Hospital, Guangzhou, China; ^2^ Department of Pharmacy, Guangdong Second Provincial General Hospital, Guangzhou, China; ^3^ Department of Internal Medicine, Guangzhou Panyu District Eighth People’s Hospital, Guangzhou, China; ^4^ Medical College, Shantou University, Shantou, Guangdong, China; ^5^ Department of Pharmacy, The Third Affiliated Hospital of Southern Medical University, Guangzhou, China

**Keywords:** GLP-1 receptor agonists, type 2 diabetes mellitus, gastrointestinal adverse events, Bayesian network meta-analysis, nausea, vomiting, diarrhea

## Abstract

**Objective:**

This study aims to evaluate and compare the gastrointestinal adverse effects associated with different GLP-1 receptor agonists (GLP-1RAs) and multi-target analogs in patients with type 2 diabetes mellitus (T2DM) using a Bayesian network meta-analysis.

**Methods:**

A systematic search of PubMed, Embase, Cochrane Library, and ClinicalTrials.gov was conducted to identify randomized controlled trials (RCTs) assessing the gastrointestinal adverse events of GLP-1RAs in T2DM patients. Inclusion criteria included adult patients with confirmed T2DM receiving any GLP-1RA, with the outcomes focused on gastrointestinal adverse events such as nausea, vomiting, diarrhea, constipation, dyspepsia, and reduced appetite. Bayesian network meta-analysis was used to calculate the odds ratios (ORs) and 95% confidence intervals (CIs) for the comparison of gastrointestinal side effects among different GLP-1RAs.

**Results:**

A total of 48 RCTs involving 27,729 participants were included in the analysis. The overall incidence of gastrointestinal adverse events was 11.66%, with nausea being the most frequent (21.49%) and reduced appetite the least frequent (5.49%). Tirzepatide had the highest risk of inducing nausea and diarrhea, while dulaglutide and lixisenatide had the lowest risks. Exenatide exhibited the highest incidence of vomiting, while dulaglutide showed a lower risk. Semaglutide demonstrated a significantly higher risk of diarrhea compared to other GLP-1RAs.

**Conclusion:**

This study highlights significant differences in the gastrointestinal adverse event profiles of various GLP-1RAs. Tirzepatide exhibited the highest risk of gastrointestinal side effects, whereas dulaglutide and exenatide showed relatively better tolerability. These findings provide valuable insights for clinicians to make informed treatment decisions, emphasizing the importance of individualized therapy based on patient tolerance.

**Systematic Review Registration:**

CRD42024592308.

## 1 Introduction

According to the International Diabetes Federation, the global population with diabetes reached 537 million by 2021, with 90% of cases attributed to type 2 diabetes (T2DM) (Ahmad et al., 2022). In 2021, diabetes was responsible for 6.7 million deaths, and it is projected that by 2045, the number of individuals with diabetes will increase to 783 million, highlighting the growing global burden of T2DM over the coming decades (Sun et al., 2022). Early diagnosis and effective treatment strategies are crucial for mitigating the risks of microvascular and macrovascular complications, as well as reducing mortality associated with T2DM.

In recent years, glucagon-like peptide-1 receptor agonists (GLP-1RAs) have gained attention not only for their role in glycemic control in T2DM but also for their potential in weight management. Increasing evidence indicates that GLP-1RAs can significantly reduce body weight, particularly in overweight or obese patients with T2DM. Semaglutide, in particular, has demonstrated superior efficacy in weight reduction across several clinical trials, driving increased interest and investment in this therapeutic area (Pagidipati, 2024). In 2021, the U.S. Food and Drug Administration (FDA) approved semaglutide for obesity management, marking a new milestone in the application of GLP-1RAs for weight loss (Jordan et al., 2024). Similarly, tirzepatide, a novel dual agonist targeting both GLP-1 and glucose-dependent insulinotropic polypeptide (GIP) receptors, has emerged as a potent therapeutic option, demonstrating remarkable efficacy in glycemic control and weight reduction in T2DM patients (Frías et al., 2021). Its unique dual mechanism enhances incretin effects, offering superior outcomes compared to traditional GLP-1RAs in recent trials, though its gastrointestinal tolerability profile warrants further scrutiny. This dual functionality makes GLP-1RAs an ideal therapeutic option for patients with T2DM and coexisting obesity. However, despite their notable efficacy, gastrointestinal side effects remain a significant limitation in their clinical use.

Gastrointestinal adverse events, such as nausea, vomiting, constipation, diarrhea, and dyspepsia, are the most common side effects associated with GLP-1RA therapy. These effects are closely related to the activation of GLP-1 receptors in both central and peripheral pathways, often leading to reduced treatment adherence or even discontinuation, which can compromise therapeutic outcomes (Tobaiqy, 2024). Although numerous studies have evaluated the efficacy of GLP-1RAs, there remains a lack of comparative data on the gastrointestinal risk profiles of different GLP-1RAs. Some studies have utilized traditional meta-analyses to assess the adverse effects of individual GLP-1RAs. However, conventional meta-analyses are typically limited to pairwise comparisons and are unable to simultaneously evaluate multiple GLP-1RAs, which restricts their clinical applicability.

Bayesian network meta-analysis offers a solution to this limitation by enabling the simultaneous comparison of multiple treatment options, providing a more precise evaluation of the differences between drugs (Wang et al., 2024). Additionally, this method allows for the integration of indirect evidence, thus offering a more comprehensive basis for clinical decision-making. To date, no network meta-analysis has systematically compared the gastrointestinal adverse events associated with different GLP-1RAs. Therefore, this study aims to evaluate the relative risks of gastrointestinal side effects associated with various GLP-1RAs in patients with T2DM through a Bayesian network meta-analysis, providing clinicians with robust evidence to inform treatment decisions.

## 2 Materials and methods

This study was designed and conducted according to the registration requirements of the PROSPERO database (CRD42024592308) and aims to systematically evaluate the gastrointestinal adverse effects of different GLP-1 receptor agonists (GLP-1RAs) in patients with type 2 diabetes mellitus (T2DM). The study was approved by the Ethics Committee of Guangzhou Eighth People’s Hospital of Panyu District, ensuring that all procedures were performed in accordance with the ethical standards outlined by the institutional research committee and the 1964 Helsinki Declaration and its later amendments or comparable ethical standards.

### 2.1 Literature search

To ensure the inclusion of high-quality randomized controlled trials (RCTs), a comprehensive literature search was conducted across PubMed, Embase, Cochrane Library, and ClinicalTrials.gov databases, covering the period from database inception to 1 November 2024. The search strategy targeted studies involving GLP-1 receptor agonists (e.g., exenatide, liraglutide, dulaglutide, lixisenatide, benaglutide, loxenatide, semaglutide) and multi-target receptor agonists (e.g., tirzepatide), focusing on gastrointestinal adverse events (e.g., nausea, vomiting, diarrhea, constipation, dyspepsia, and reduced appetite) in patients with type 2 diabetes mellitus (T2DM) (Supplementary Material). Search terms included relevant keywords and controlled vocabulary (e.g., MeSH and Emtree terms) for T2DM, GLP-1 receptor agonists, multi-target agonists, and gastrointestinal outcomes, combined with terms for randomized controlled trials. Studies involving type 1 diabetes, pregnancy, or breastfeeding were excluded.

#### 2.1.1 Inclusion criteria

Studies were eligible for inclusion if they met the following criteria:

a. Patients aged ≥18 years with a confirmed diagnosis of type 2 diabetes mellitus (T2DM) receiving GLP-1RA treatment.
b. The intervention group received any of the specified GLP-1RAs, while the control group could consist of another GLP-1RA or placebo, with no restrictions on dosage, frequency, or mode of administration.
c. The outcomes included any gastrointestinal adverse events, such as nausea, vomiting, dyspepsia, constipation, diarrhea, and loss of appetite.

#### 2.1.2 Exclusion criteria

Studies were excluded if they met any of the following criteria:

a. Patients with pre-existing gastrointestinal diseases.
b. Patients with type 1 diabetes.
c. Pregnant or breastfeeding women.
d. Patients with severe cardiovascular disease or other metabolic disorders, excluding diabetes.

### 2.2 Literature screening and data extraction

Two independent researchers manually screened the titles and abstracts of all studies to ensure relevance to the study topic, resolving discrepancies through discussion to maintain rigor in the selection process. Subsequently, the full texts of the included studies were analyzed by both researchers independently, ensuring impartiality and consistency. In case of disagreements, they were resolved through consensus or arbitration by a third researcher. Data extraction was performed by one researcher using a predefined data extraction form, and the extracted data were independently verified by another researcher to ensure accuracy and validity.

The extracted data included the following:

a. Study characteristics: study topic, first author, publication year, clinical trial registration number, GLP-1RA drug name, and dosage;
b. Baseline characteristics: sample size and median age of study participants;
c. Outcome measures: all outcome variables related to gastrointestinal adverse events;
d. Study quality assessment information: data extracted according to predefined quality assessment criteria.

Adverse events were extracted as reported in the primary studies without attempting to reclassify or standardize the terms beyond what was provided in the original publications. We acknowledge that classification of gastrointestinal AEs, such as nausea, vomiting, diarrhea, constipation, dyspepsia, and reduced appetite, may vary across studies, which could introduce some heterogeneity in the reported incidences.

### 2.3 Statistical analysis

Bayesian network meta-analysis was performed using Stata 14 software (STATA Corporation, College Station, TX), with a significance level set at *P* < 0.05. Based on the Markov Chain Monte Carlo (MCMC) method (Gelman and Rubin, 1996), a generalized linear model with random effects was employed to conduct the Bayesian network meta-analysis. A consistency model was used to calculate the effect sizes as odds ratios (ORs) with their 95% confidence intervals (95% CI).

To obtain the posterior distribution, four chains were run simultaneously, with each chain undergoing 50,000 burn-in iterations and 100,000 inference iterations. The Gelman-Rubin diagnostic method was used to assess the convergence of the model, and convergence was further evaluated by plotting density and area graphs (Brooks and Gelman, 1998). A network plot was generated to summarize the evidence across different studies, and a comparison-adjusted funnel plot was used to evaluate the risk of publication bias.

Additionally, the surface under the cumulative ranking curve (SUCRA) was calculated to estimate the probability of different GLP-1RAs causing gastrointestinal adverse events and to rank the safety of the drugs.

## 3 Results

### 3.1 Literature search and quality assessment

**FIGURE 1:**
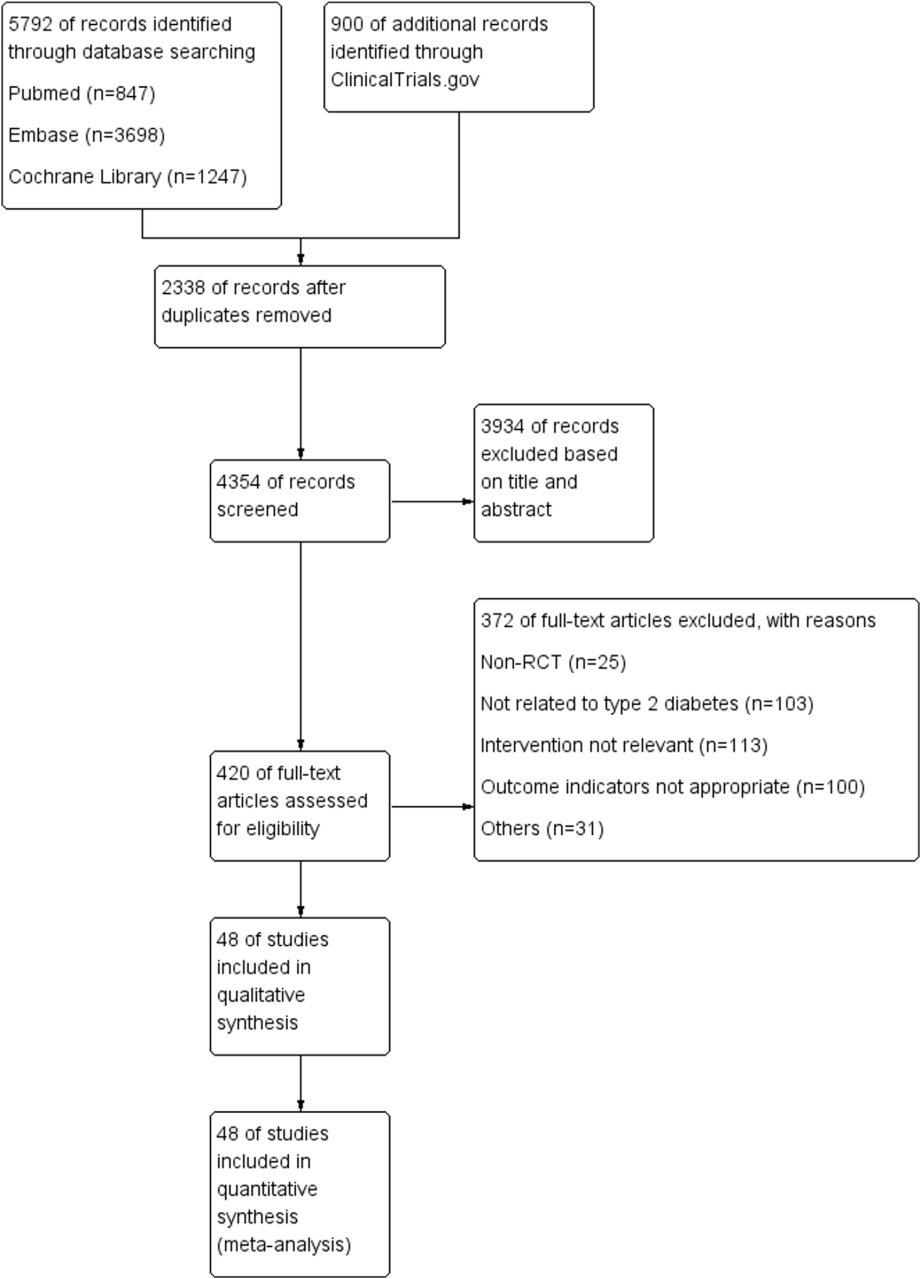
Flowchart of literature search and screening.

A total of 4,354 relevant studies were identified through a systematic search. After an initial screening of titles and abstracts, 420 studies were selected for full-text review. Following further evaluation, 372 studies were excluded for not meeting the inclusion criteria. Ultimately, 48 studies were included in the analysis, covering 7 different GLP-1RAs (Figure 1).

### 3.2 Basic characteristics of the included studies

A total of 48 randomized controlled trials (RCTs) were included in this analysis(Frías et al., 2021; Ahmann et al., 2015; Ahmann et al., 2018; Ahrén et al., 2013; Ahrén et al., 2017; Bolli et al., 2014; Buse et al., 2004; Buse et al., 2009; Buse et al., 2011; Buse et al., 2013; Capehorn et al., 2020; Apovian et al., 2010; Davies et al., 2017; DeFronzo et al., 2005; Dungan et al., 2014; Dungan et al., 2016; Ferdinand et al., 2014; Gao et al., 2020; Russell-Jones et al., 2009; Kadowaki et al., 2011; Kendall et al., 2005; Korsatko et al., 2018; Lind et al., 2015; Lingvay et al., 2018; Liutkus et al., 2010; Ludvik et al., 2018; Miras et al., 2019; Miyagawa et al., 2015; Mosenzon et al., 2019; Nauck et al., 2014; Pinget et al., 2013; Pozzilli et al., 2017; Pratley et al., 2018; Pratley et al., 2019; Rodbard et al., 2018; Rosenstock et al., 2013; Rosenstock et al., 2014; Seino et al., 2012; Shaman et al., 2022; Sorli et al., 2017; Wysham et al., 2014; Yabe et al., 2020; Yamada et al., 2020; Yang et al., 2018; Yu Pan et al., 2014; Zinman et al., 2019b), with a combined sample size of 27,729 participants. Among them, 11 studies were related to liraglutide, 16 to semaglutide, 12 to exenatide, 8 to lixisenatide, 10 to dulaglutide, 1 to loxenatide, and 1 to tirzepatide. The average age of the patients was 56.59 ± 4.96 years, with 52.71% ± 11.45% being male. The average weight was 88.6 ± 11.9 kg, and the body mass index (BMI) was 31.7 ± 3.0 kg/m^2^. The duration of T2DM was 8.6 ± 3.4 years, baseline glycated hemoglobin (HbA1c) was 8.14% ± 0.29% (65.16 ± 3.11 mmol/mol), and fasting blood glucose was 9.84 ± 6.98 mmol/L.

### 3.3 Quality assessment of included studies

The evaluation of the included studies indicated a low risk of bias in general. In key areas such as randomization, outcome measurement, selective reporting, and handling of incomplete data, most studies exhibited a low risk of bias, reflecting the rigor of their study designs and data management. However, certain studies demonstrated a risk of bias in specific critical aspects.

**FIGURE 2:**
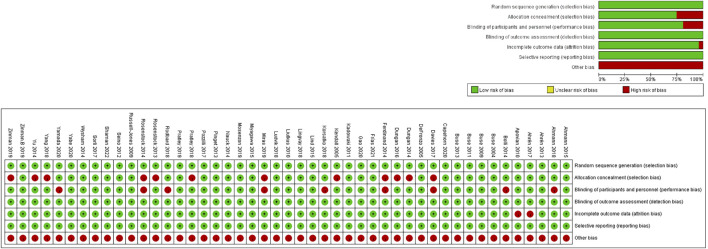
Quality evaluation of included documents.

In particular, 14 studies (31%) were found to have a high risk of bias in allocation concealment, suggesting that these studies may not have fully implemented proper allocation concealment during randomization, potentially leading to imbalanced group assignments or selection bias. Additionally, 8 studies (18%) exhibited a high risk of bias related to blinding of participants (Figure 2), indicating that blinding was not adequately maintained for participants or intervention administrators, which may have impacted the assessment of intervention effects. Despite these risks, the overall quality of these studies remained high in other important domains.

**FIGURE 3:**
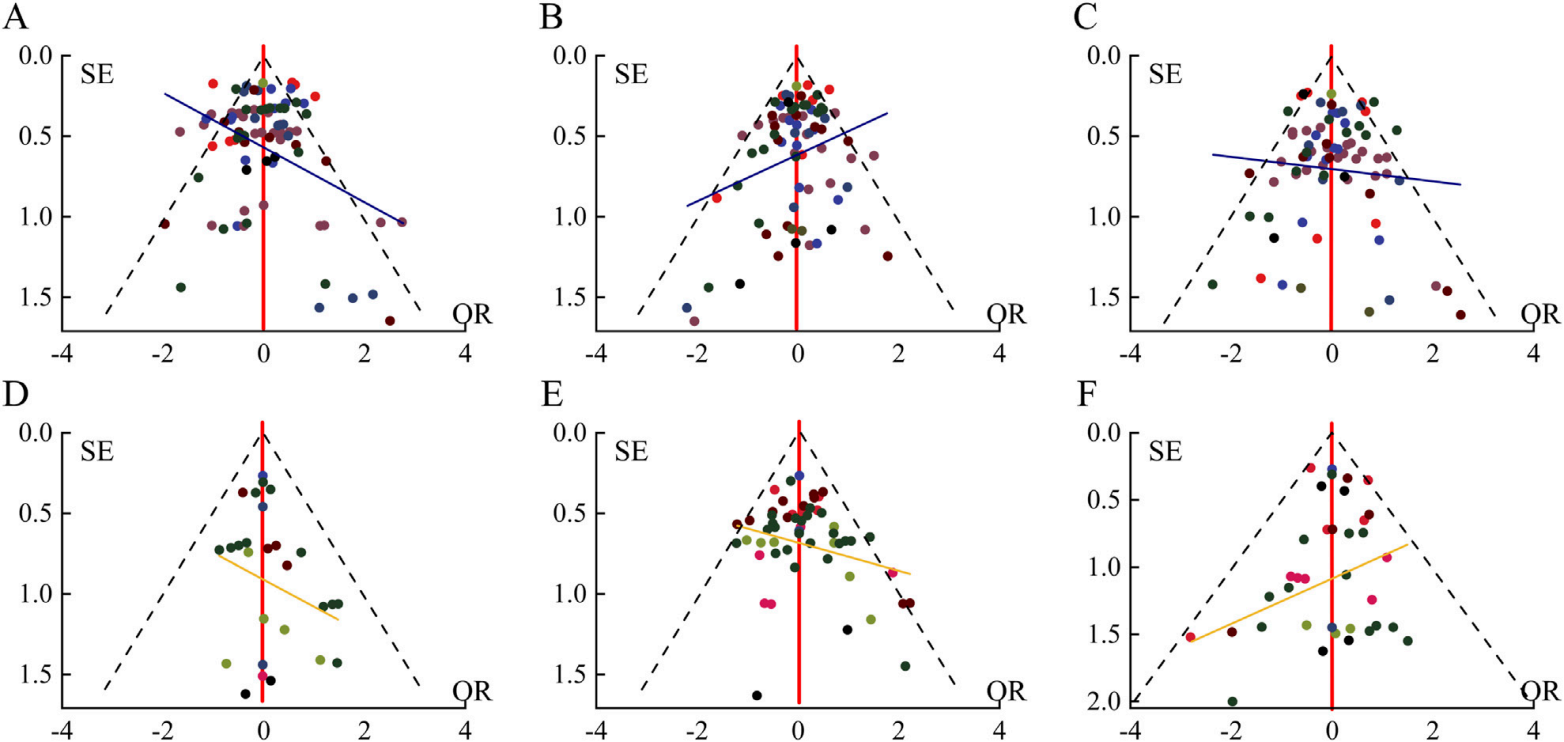
Funnel Plots Illustrating Publication Bias in Gastrointestinal Adverse Events Induced by GLP-1 Receptor Agonists in Type 2 Diabetic Patients. Note: Funnel plots for the analysis of publication bias in gastrointestinal adverse events associated with GLP-1 receptor agonists. Each panel represents a specific adverse event: **(A)** Nausea, **(B)** Diarrhea, **(C)** Vomiting, **(D)** Indigestion, **(E)** Constipation, **(F)** Appetite loss. The x-axis represents the effect size (QR), and the y-axis shows the standard error (SE) of the studies included in the Bayesian network meta-analysis. Symmetry in the funnel plot indicates low risk of publication bias, while asymmetry suggests potential bias. Different colored points represent individual studies contributing to the meta-analysis. The dashed lines indicate the 95% confidence interval.

The analysis of the funnel plot (Figure 3) showed no significant asymmetry, indicating that there was no notable small-study effect or publication bias, further supporting the robustness of the findings. Furthermore, all included studies adhered to the predefined quality standards, ensuring the credibility and reproducibility of this meta-analysis.

In conclusion, while some studies demonstrated a high risk of bias in allocation concealment and blinding, their quality in other critical aspects was maintained. As a result, the overall quality of the included studies is acceptable, providing a solid foundation for subsequent analyses.

### 3.4 Incidence of gastrointestinal adverse events

**FIGURE 4:**
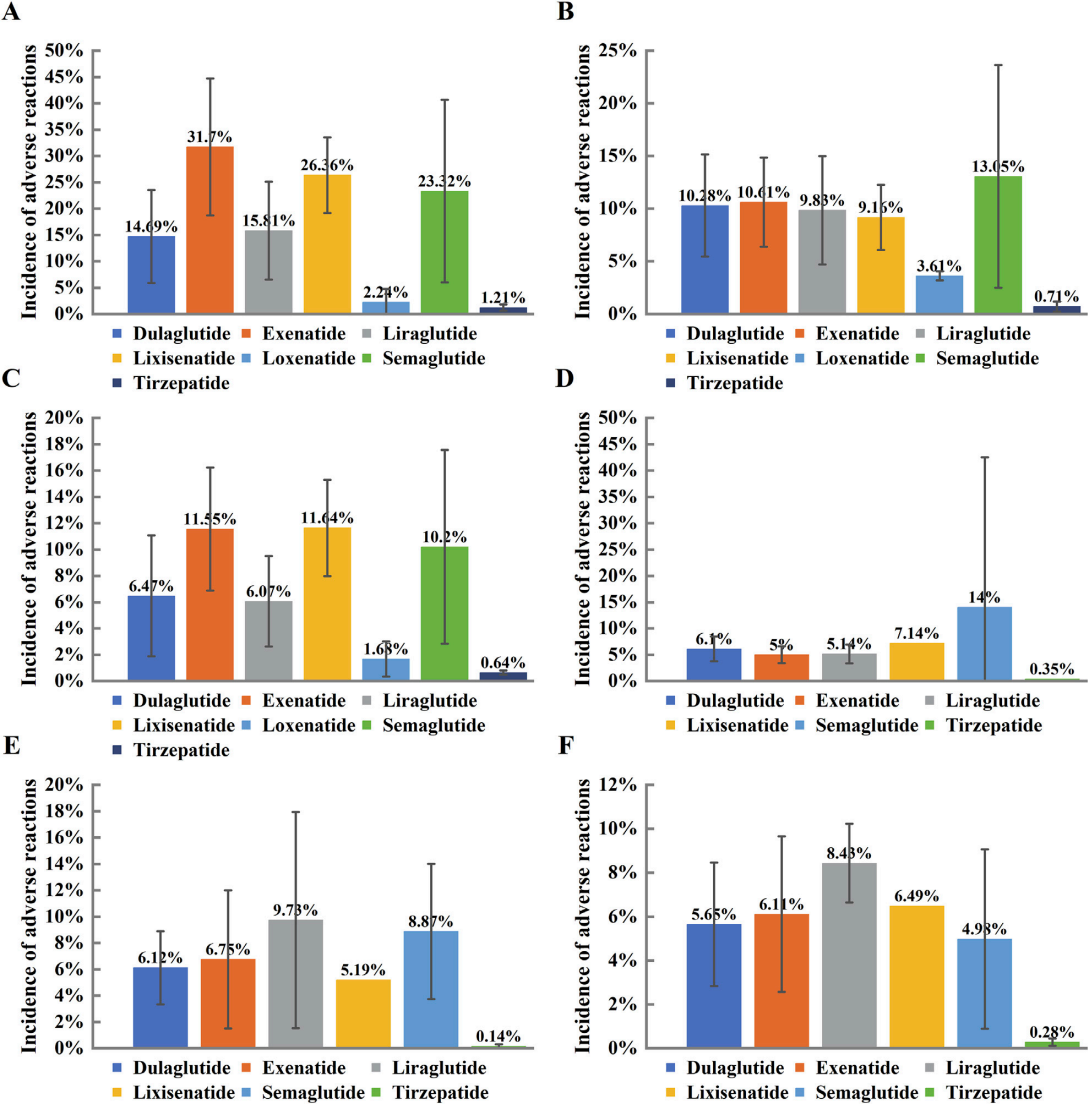
Incidence of Gastrointestinal Adverse Reactions in Type 2 Diabetic Patients Treated with GLP-1 Receptor Agonists. Note: Bar charts representing the incidence of various gastrointestinal adverse reactions in Type 2 diabetic patients treated with different GLP-1 receptor agonists, including Dulaglutide, Exenatide, Liraglutide, Lixisenatide, Semaglutide, and Tirzepatide. Each panel corresponds to a specific adverse event: **(A)** Nausea, **(B)** Diarrhea, **(C)** Vomiting, **(D)** Indigestion, **(E)** Constipation, **(F)** Appetite loss. The x-axis shows the different GLP-1 receptor agonists, and the y-axis indicates the incidence of the respective adverse reaction (%). Error bars represent the 95% confidence intervals. The results highlight varying rates of adverse reactions across different drugs, with Tirzepatide and Liraglutide showing higher incidence rates in several categories.

Among all the included studies, the overall incidence of gastrointestinal adverse events caused by GLP-1RAs was 11.66%. Specifically, the incidence rates of six common gastrointestinal adverse events were as follows: nausea (21.49%), diarrhea (10.62%), vomiting (9.10%), dyspepsia (8.67%), constipation (7.92%), and decreased appetite (5.49%). These data reflect the prevalence of gastrointestinal adverse events during GLP-1RA treatment and highlight the differences in incidence rates across various types of adverse events (Figure 4).

Nausea was the most common gastrointestinal adverse event, reported in almost all GLP-1RA treatment regimens. The highest incidence of nausea was observed with exenatide, at 31.70%, demonstrating a significantly higher risk of nausea compared to other GLP-1RAs. Although other drugs had lower nausea incidence rates, this adverse event still has clinical significance. Diarrhea ranked second with an incidence of 10.62%. Semaglutide and dulaglutide were more likely to cause diarrhea, suggesting that patient tolerance should be considered when selecting GLP-1RA treatments. In contrast, other GLP-1RAs had relatively lower risks of causing diarrhea.

Vomiting had an incidence of 9.10%, which is another important adverse event to monitor. Notably, exenatide also had a higher incidence of vomiting, which may be related to its higher incidence of nausea. The incidence of dyspepsia was 8.67%. Although lower than nausea and vomiting, dyspepsia may still lead to treatment discontinuation in some patients. Dulaglutide and liraglutide had lower risks of dyspepsia, indicating that they may offer better gastrointestinal tolerance.

Constipation had an incidence of 7.92%, which was slightly lower compared to other gastrointestinal adverse events. Notably, tirzepatide had the lowest incidence of constipation, at only 0.14%, indicating that this drug poses minimal risk of constipation. The incidence of decreased appetite was 5.49%, which was relatively low. However, semaglutide and liraglutide showed higher rates of decreased appetite, which may be associated with their weight loss effects (Blundell et al., 2017).

### 3.5 Network meta-analysis results

**FIGURE 5:**
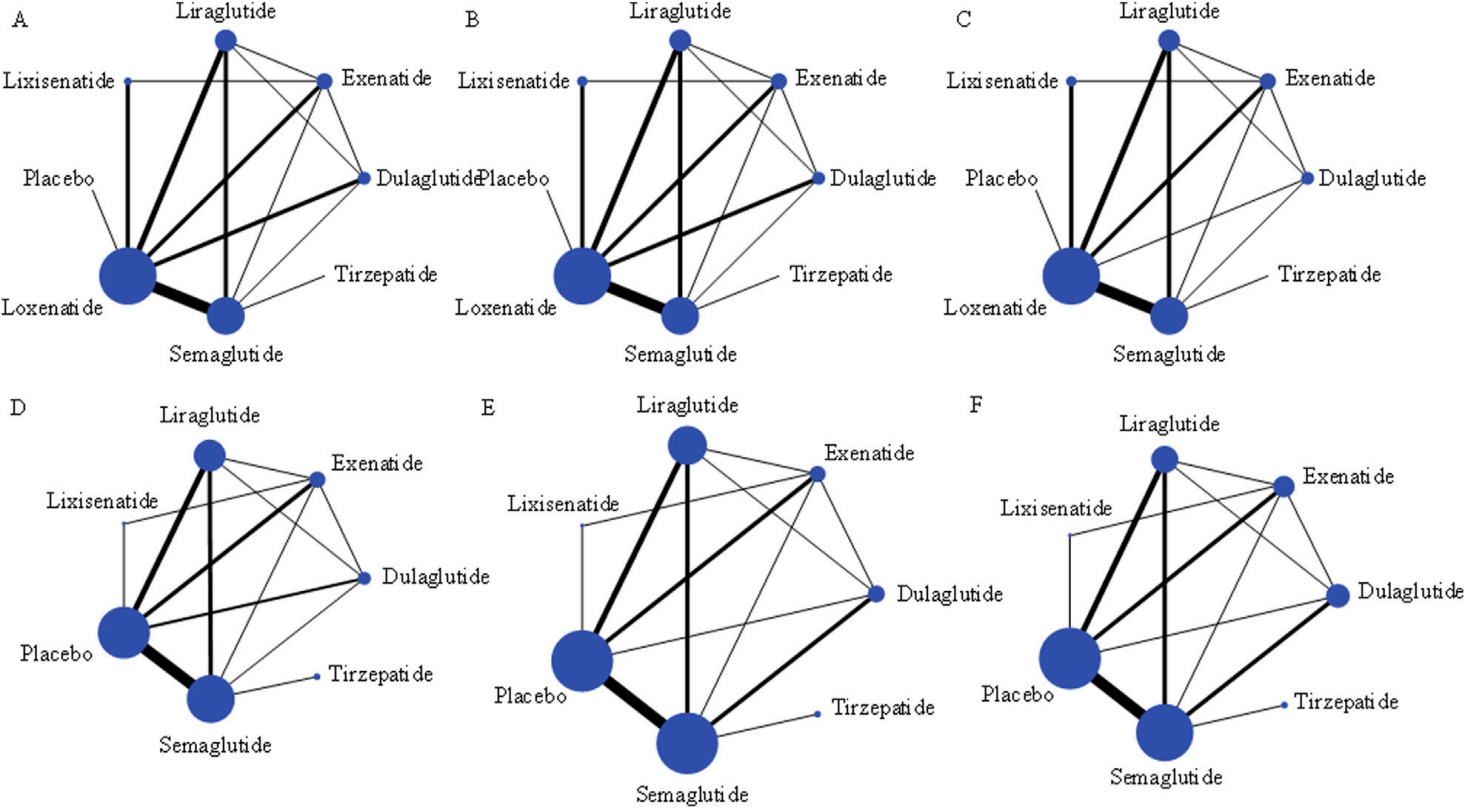
Network Meta-Analysis of Gastrointestinal Adverse Reactions in Type 2 Diabetic Patients Treated with GLP-1 Receptor Agonists. Note: Bubble plots representing the network meta-analysis of gastrointestinal adverse reactions in Type 2 diabetic patients treated with different GLP-1 receptor agonists. The size of the bubbles corresponds to the sample size or weight of each study within the network. Panels display specific adverse reactions: **(A)** Nausea, **(B)** Diarrhea, **(C)** Vomiting, **(D)** Indigestion, **(E)** Constipation, **(F)** Appetite loss. Each bubble represents a comparison between different GLP-1 receptor agonists, with larger bubbles indicating a greater impact or more substantial evidence. The plots visualize the connections and comparisons across multiple treatments within the network, highlighting the incidence of each adverse reaction.

**FIGURE 6:**
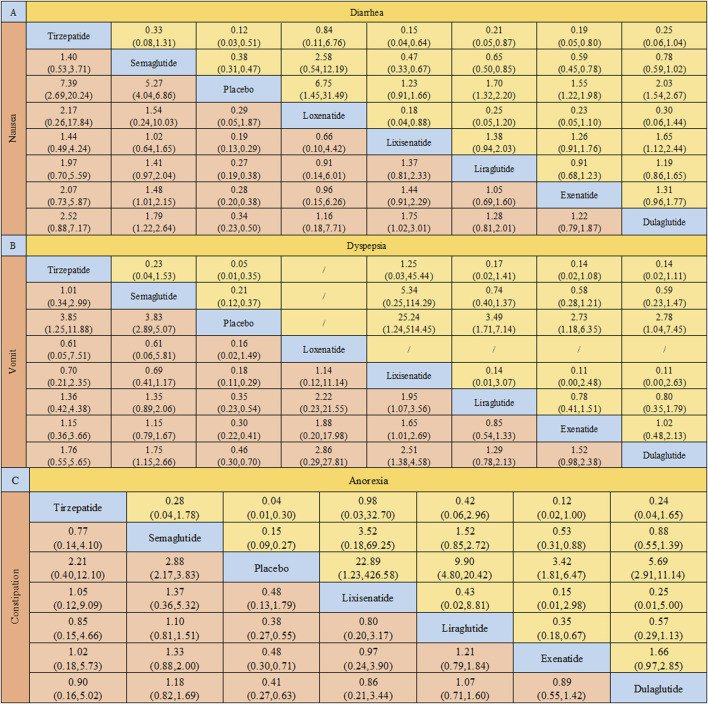
Pairwise Comparisons of Gastrointestinal Adverse Reactions Induced by GLP-1 Receptor Agonists in Type 2 Diabetic Patients. Note: Heatmap of pairwise comparisons showing the incidence of gastrointestinal adverse reactions in Type 2 diabetic patients treated with different GLP-1 receptor agonists. The comparisons are made between Tirzepatide, Semaglutide, Lixisenatide, Liraglutide, Exenatide, Dulaglutide, and placebo. Panels represent the following adverse reactions: **(A)** Nausea and Diarrhea, **(B)** Vomiting and Indigestion (Dyspepsia), **(C)** Constipation and Appetite Loss (Anorexia). Each cell contains the odds ratio (OR) and 95% confidence interval (CI) for the comparison, with color coding indicating the magnitude of the effect. Blue cells represent comparisons with significant effects favoring the drug listed in the column, while red cells highlight significant adverse effects for the drug in the row. Yellow cells show non-significant comparisons. The heatmap allows for a visual comparison of how each drug performs against others in terms of inducing gastrointestinal side effects.

The network relationship analysis indicates significant differences in the incidence of gastrointestinal adverse events across different GLP-1RAs (Figure 5), with the analysis results as follows (Figure 6):

Nausea (Kendall et al., 2005; Lingvay et al., 2018; Liutkus et al., 2010; Ludvik et al., 2018; Miyagawa et al., 2015; Mosenzon et al., 2019; Pinget et al., 2013; Pozzilli et al., 2017; Pratley et al., 2018; Pratley et al., 2019; Rodbard et al., 2018): Compared with placebo, the risk of nausea with loxenatide was not significantly increased (OR = 0.29, 95% CI: 0.05–1.87), while other GLP-1RAs significantly increased the incidence of nausea (P < 0.05). Comparisons between drugs showed that exenatide (OR = 1.48, 95% CI: 1.01–2.15) and dulaglutide (OR = 1.79, 95% CI: 1.22–2.64) had a significantly lower risk of nausea compared to semaglutide. Additionally, the risk of nausea with lixisenatide was higher than that of dulaglutide (OR = 1.75, 95% CI: 1.02–3.01), with an overall incidence of 21.49%, estimated at 31.7% for exenatide, 21.5% for semaglutide, 25% for tirzepatide, 20% for lixisenatide, 15% for liraglutide, and 10% for dulaglutide.

Diarrhea (Frías et al., 2021; Ahmann et al., 2015; Ahmann et al., 2018; Ahrén et al., 2013; Bolli et al., 2014; Buse et al., 2004; Buse et al., 2009; Buse et al., 2011; Buse et al., 2013; Capehorn et al., 2020; Apovian et al., 2010; Davies et al., 2017; DeFronzo et al., 2005; Dungan et al., 2014; Dungan et al., 2016; Ferdinand et al., 2014; Gao et al., 2020; Russell-Jones et al., 2009; Kadowaki et al., 2011; Kendall et al., 2005; Korsatko et al., 2018): Compared with placebo, lixisenatide showed no significant difference in the risk of diarrhea (OR = 1.23, 95% CI: 0.91–1.66) However, compared to tirzepatide, the risk of diarrhea was significantly lower with lixisenatide, liraglutide, and exenatide (OR = 0.15, 95% CI: 0.04–0.64; OR = 0.21, 95% CI: 0.05–0.87; OR = 0.19, 95% CI: 0.05–0.80). Similarly, compared to semaglutide, the risk of diarrhea was also significantly lower with lixisenatide, liraglutide, and exenatide (OR = 0.47, 95% CI: 0.33–0.67; OR = 0.65, 95% CI: 0.50–0.85; OR = 0.59, 95% CI: 0.45–0.78), with an overall incidence of 10.62%, estimated at 15% for tirzepatide, 10.6% for semaglutide, 10% for loxenatide, 8% for dulaglutide, 7% for exenatide, and 5% for lixisenatide.

Vomiting (Frías et al., 2021; Ahmann et al., 2015; Ahmann et al., 2018; Ahrén et al., 2013; Bolli et al., 2014; Buse et al., 2004; Buse et al., 2009; Buse et al., 2011; Buse et al., 2013; Capehorn et al., 2020; Apovian et al., 2010; Davies et al., 2017; DeFronzo et al., 2005; Dungan et al., 2014; Ferdinand et al., 2014; Gao et al., 2020; Russell-Jones et al., 2009; Kadowaki et al., 2011; Kendall et al., 2005; Miras et al., 2019; Wysham et al., 2014; Yabe et al., 2020; Yu Pan et al., 2014; Zinman et al., 2019a): The risk of vomiting with loxenatide was not significantly increased compared to placebo (OR = 0.16, 95% CI: 0.02–1.49), while other GLP-1RAs significantly increased the incidence of vomiting. Comparisons between drugs showed that semaglutide had a significantly higher risk of vomiting compared to dulaglutide (OR = 1.75, 95% CI: 1.15–2.66). Additionally, lixisenatide showed a significantly higher risk of vomiting compared to liraglutide (OR = 1.95, 95% CI: 1.07–3.56), exenatide (OR = 1.65, 95% CI: 1.01–2.69), and dulaglutide (OR = 2.51, 95% CI: 1.38–4.58), with an overall incidence of 9.10%, estimated at 10% for lixisenatide, 9% for loxenatide and tirzepatide, 9% for semaglutide and exenatide, and 5% for dulaglutide.

Dyspepsia (Frías et al., 2021; Ahmann et al., 2015; Ahmann et al., 2018; Ahrén et al., 2013; Buse et al., 2009; Buse et al., 2011; Buse et al., 2013; Davies et al., 2017; Dungan et al., 2014; Russell-Jones et al., 2009): Compared to placebo, all GLP-1RAs significantly increased the risk of dyspepsia (P < 0.05), suggesting an increased likelihood of developing dyspepsia during treatment with GLP-1RAs. The overall incidence was 8.67%, estimated at 10% for tirzepatide, 8% for lixisenatide and semaglutide, 7% for liraglutide and dulaglutide, and 6% for exenatide.

Constipation (Frías et al., 2021; Ahmann et al., 2018; Buse et al., 2004; Buse et al., 2009; Buse et al., 2011; Buse et al., 2013; Capehorn et al., 2020; Davies et al., 2017; Dungan et al., 2014; Kadowaki et al., 2011): Compared to placebo, neither tirzepatide (OR = 2.21, 95% CI: 0.401–2.10) nor lixisenatide (OR = 0.48, 95% CI: 0.13–1.79) showed a significant difference in the risk of constipation. Additionally, there were no significant differences in the risk of constipation between GLP-1RAs (P > 0.05). The overall incidence was 7.92%, estimated at 8% for semaglutide, 7% for liraglutide, 6% for dulaglutide, and 0.14% for tirzepatide.

Reduced Appetite (Frías et al., 2021; Ahmann et al., 2018; Buse et al., 2013; Kadowaki et al., 2011; Miyagawa et al., 2015; Wysham et al., 2014; Yabe et al., 2020; Yamada et al., 2020; Zinman et al., 2019a): Compared to placebo, all GLP-1RAs significantly reduced appetite (P < 0.05). Among the drugs, semaglutide (OR = 0.53, 95% CI: 0.31–0.88) and liraglutide (OR = 0.35, 95% CI: 0.18–0.67) had a significantly higher risk of reduced appetite compared to exenatide with an overall incidence of 5.49%, estimated at 10% for tirzepatide, 6% for lixisenatide, 5% for liraglutide and semaglutide, and 4% for dulaglutide.

### 3.6 Inconsistency testing and ranking of GLP-1RA-induced gastrointestinal adverse events

A node-splitting analysis was used to assess the consistency between direct and indirect evidence. The results showed no significant differences between treatment groups (P > 0.05), indicating that the consistency model was appropriate and the data were reliable. Based on this model, the ranking of the risk of gastrointestinal adverse events induced by GLP-1RAs showed notable differences.

Tirzepatide had the highest risk of inducing nausea and diarrhea, while dulaglutide and lixisenatide had the lowest risks for these adverse events. Lixisenatide had the highest risk of inducing vomiting, while dulaglutide had the lowest. For dyspepsia, tirzepatide posed the highest risk, whereas exenatide had the lowest. Semaglutide had the highest risk of inducing constipation, while exenatide had the lowest. Tirzepatide was associated with the highest risk of appetite reduction, while exenatide had the lowest (Table 1).

**TABLE 1:** Gastrointestinal adverse reaction rankings in type 2 diabetic patients receiving GLP-1 receptor agonists.

Medicine	Nausea		Diarrhea		Vomiting		Indigestion		Constipation		Appetite loss	
	SURCA	Rank	SURCA	Rank	SURCA	Rank	SURCA	Rank	SURCA	Rank	SURCA	Rank
Dulaglutide	26.4	7	56.2	4	22.3	7	33.6	5	56.1	3	42.3	5
Exenatide	41.5	6	32.6	6	52.8	5	31.4	6	43.1	6	19.4	6
Liraglutide	46.5	5	41.0	5	38.6	6	46.9	4	65.1	2	72.1	3
Lixisenatide	74.2	3	15.1	7	86.6	1	84.8	2	48.9	5	78.9	2
Loxenatide	46.9	4	88.1	2	73.1	2	—	—	—	—	—	—
Placebo	1.4	8	1.5	8	0.9	8	1.0	7	5.5	7	0.3	7
Semaglutide	77.4	2	73.3	3	65.7	3	64.3	3	79.5	1	51.3	4
Tirzepatide	85.8	1	92.2	1	60.1	4	88.1	1	51.9	4	85.7	1

Note: SURCA, cumulative adverse reaction rate below.

## 4 Discussion

Our study spearheads a systematic assessment of gastrointestinal adverse event risks for GLP-1 receptor agonists and multi-target agonists in type 2 diabetes patients, addressing a critical gap in comparative safety data via Bayesian network meta-analysis. A total of 45 randomized controlled trials (RCTs) were included, involving 27,729 patients. The overall incidence of gastrointestinal adverse events was 11.66%, with nausea (21.49%) being the most frequently reported adverse event and reduced appetite (5.49%) the least frequent. While the overall incidence was lower than previously reported rates of 30%–50% (Jones et al., 2018), this discrepancy may be attributable to the subjective nature of symptom assessment in the included studies, as many lacked objective quantitative methods (Kong et al., 1998). Additionally, heterogeneity in study design and individual patient differences may have influenced the reported incidence rates, underscoring the need for further investigation into the evaluation of gastrointestinal adverse events in different patient populations.

The mechanisms underlying GLP-1RA-induced gastrointestinal adverse events are not yet fully understood. GLP-1RAs primarily influence gastrointestinal function by inhibiting gastric emptying and activating central GLP-1 receptors. As demonstrated in this study, short-acting GLP-1RAs, such as lixisenatide, were associated with a significantly higher risk of nausea and vomiting compared to long-acting and ultra-long-acting GLP-1RAs, such as dulaglutide and semaglutide. This finding aligns with previous studies, suggesting that short-acting agents may more readily cross the blood-brain barrier, thereby activating central GLP-1 receptors and exacerbating symptoms such as nausea and anorexia (Levin et al., 2017). Moreover, the faster metabolic rate of short-acting GLP-1RAs may induce abrupt changes in gastrointestinal function, further supporting the higher incidence of nausea and vomiting with these agents. A key finding of this study is the increased incidence of diarrhea with long-acting and ultra-long-acting GLP-1RAs, particularly semaglutide which a recent meta-analysis similarly found to have a higher diarrhea risk compared to lixisenatide, liraglutide, and exenatide, though lower than tirzepatide (risk ratios 1.66–1.80 vs. 1.81–2.18 relative to placebo) (Karagiannis et al., 2024). For exenatide, our analysis primarily reflects the short-acting formulation (Byetta^®^, dosed twice daily), which likely contributes to its higher vomiting risk due to frequent dosing and greater fluctuations in plasma concentrations. In contrast, the long-acting formulation (Bydureon^®^, dosed once weekly) utilizes a sustained-release microsphere technology, potentially reducing vomiting risk by stabilizing plasma levels, though this distinction was not separately analyzed in our meta-analysis. Diarrhea is believed to result from the effect of GLP-1RAs on intestinal motility, mediated through the vagus nerve. This effect is likely driven by the prolonged circulating half-lives of long-acting and ultra-long-acting GLP-1RAs, such as semaglutide (via fatty acid acylation) and dulaglutide (via Fc fragment fusion), which maintain sustained GLP-1 receptor stimulation and enhance intestinal motility, thereby increasing diarrhea risk. In contrast, short-acting GLP-1RAs, such as lixisenatide, require more frequent dosing due to shorter half-lives, resulting in intermittent receptor stimulation that may reduce the risk of diarrhea. This observation is corroborated by previous findings (Baraboi et al., 2011). Therefore, for patients with preexisting gastrointestinal conditions or poor gastrointestinal tolerance, short-acting GLP-1RAs may be preferable due to their intermittent receptor stimulation, potentially lowering diarrhea risk.

Tirzepatide, a dual agonist of both GLP-1 and GIP receptors, warrants special consideration in this analysis. Tirzepatide is a pivotal therapeutic option for type 2 diabetes management, chronic weight management in adults with obesity or overweight with weight-related comorbidities, and treatment of moderate to severe obstructive sleep apnea in obese adults, leveraging its dual GLP-1 and GIP receptor agonism to achieve superior glycemic control and weight reduction. It received U.S. Food and Drug Administration (FDA) approval in May 2022 for type 2 diabetes, November 2023 for chronic weight management, and December 2024 for obstructive sleep apnea, marking it as the first approved therapy for this indication. A recent meta-analysis confirms its efficacy in reducing HbA1c and body weight, though its gastrointestinal adverse event risk necessitates careful patient monitoring (Liu et al., 2025). Semaglutide and liraglutide exhibited higher rates of decreased appetite in our analysis, primarily driven by central nervous system-mediated satiety regulation, delayed gastric emptying, and modulation of appetite-regulating hormones such as ghrelin (Gupta and Sharma, 2025; Naveed et al., 2025). These mechanisms, rather than weight loss itself, are the primary contributors to appetite suppression, with weight loss as a consequence. Notably, weight loss further reinforces appetite reduction through decreased ghrelin levels, establishing a bidirectional feedback loop that enhances therapeutic outcomes (Blundell et al., 2017). The role of GIP receptor agonism in tirzepatide’s gastrointestinal profile remains debated. While earlier evidence suggested GIP agonism may exacerbate GI adverse events (Min and Bain, 2021), recent preclinical and clinical studies indicate it may reduce nausea and vomiting, though its effect on diarrhea is less pronounced. In animal models, GIP receptor agonists significantly attenuated GLP-1R agonist-induced nausea and emesis while maintaining glycemic and weight loss benefits (Borner et al., 2021). A clinical study further supports that co-administration of GIP receptor agonists with GLP-1RAs reduces nausea and vomiting incidence, potentially via neuronal interactions in the hypothalamus and brainstem, though diarrhea rates remain comparable (Knop et al., 2024). These findings suggest tirzepatide’s dual agonism may contribute to its favorable GI profile in some patients, warranting further investigation to reconcile these conflicting perspectives. Our findings that tirzepatide has the highest risk of nausea and diarrhea, dulaglutide and lixisenatide the lowest, and exenatide the highest vomiting risk with dulaglutide showing a lower risk, are consistent with a recent meta-analysis reporting higher diarrhea risk for tirzepatide (risk ratio 1.81–2.18) than semaglutide (1.66–1.80) versus placebo, as well as trial data showing elevated nausea with tirzepatide and higher vomiting with exenatide’s short-acting formulation (Karagiannis et al., 2024).

Effective management of gastrointestinal adverse events associated with GLP-1 receptor agonists and multi-target agonists is critical to optimize their therapeutic application in type 2 diabetes management. Gradual dose escalation can significantly mitigate the frequency and severity of symptoms such as nausea and diarrhea, as it allows patients to adapt to therapy over time (Huang et al., 2025; Rodriguez et al., 2025). This approach facilitates physiological adaptation to GLP-1 and GIP receptor stimulation, enabling patients to develop tolerance to gastrointestinal symptoms over weeks to months, thereby reducing their intensity. Patient education emphasizing the transient nature of these side effects, particularly for high-risk agents like tirzepatide and semaglutide, can enhance adherence by setting realistic expectations. Dietary modifications, such as adjusting meal timing or composition, may further alleviate discomfort during the initial treatment phase. For patients with persistent or intolerable symptoms, switching to agents with lower gastrointestinal risk profiles, such as dulaglutide, which demonstrated reduced nausea incidence in our analysis, represents a viable strategy. Emerging evidence suggests that dual GLP-1/GIP agonists like tirzepatide may offer a more favorable gastrointestinal profile in some patients, potentially improving adherence (Kim et al., 2025). Clinicians must also remain vigilant for rare but serious complications, such as bowel obstruction, particularly in patients with preexisting motility disorders, although these risks may be overstated when comorbidities are considered (Jones and Cappola, 2025; Kałas et al., 2025; Gmehlin et al., 2025). Additionally, addressing the psychological impact of these adverse events is essential, as GLP-1RAs may influence emotional regulation, necessitating a holistic approach to patient care (Lu et al., 2025). Regular follow-up and supportive care are recommended to monitor and manage ongoing symptoms effectively (Narayan et al., 2025).

It should be noted that this study did not conduct subgroup analyses based on dosage and administration routes, which may limit the comprehensive assessment of GLP-1RA safety. The variability in trial durations, ranging from 3 to 12 weeks across the included studies, and differences in the number of titration steps for each drug may further influence the reported incidence of gastrointestinal adverse events, particularly for agents like tirzepatide, where nausea risk escalates with higher doses (e.g., OR = 4.36, 95% CI: 3.42–5.61 at 10 mg) (Liu et al., 2025). Previous studies have shown that the incidence of gastrointestinal adverse events varies depending on GLP-1RA dosage, particularly at higher doses, where nausea and vomiting are more prevalent. Additionally, baseline characteristics of patients, such as age, BMI, and renal function, may significantly impact the occurrence of GLP-1RA-induced adverse events. Other factors, including drop-out rates due to GI AEs, use of anti-emetics or other rescue therapies, specific dosing regimens (e.g., semaglutide’s approved doses of 0.5–1.0 mg for T2D vs. 2.4 mg for weight management), number of titration steps, route of administration (subcutaneous vs. oral), and pharmacokinetic properties (e.g., absorption rates, peak-to-trough ratios, and compound half-lives), were not analyzed in this study. These factors may significantly influence GI AE incidence, as higher doses, oral administration, or rapid absorption may exacerbate nausea and vomiting, while gradual titration can mitigate such effects. Sex distribution was not analyzed in our study, which is a limitation given evidence that females may be more susceptible to nausea and vomiting induced by GLP-1RAs, potentially due to hormonal influences or differences in gastric emptying rates. These factors were not explored in detail in this study. A potential limitation of this study is the variability in adverse event classification across different trials. Terms such as nausea, loss of appetite, and feeling full or bloated may overlap in some studies, and vomiting could be confounded with dyspepsia or gastroesophageal reflux disease (GERD). This variability could affect the comparability of AE incidences between different GLP-1RAs. However, our network meta-analysis included a large number of studies (n = 45), and the inconsistency testing using node-splitting analysis showed no significant differences between direct and indirect evidence (P > 0.05), suggesting that the data are consistent and reliable for the purposes of this analysis. Nonetheless, future studies with standardized AE reporting, such as using the Medical Dictionary for Regulatory Activities (MedDRA), would be beneficial to confirm our findings and enhance comparability across trials. Thus, further large-scale prospective studies are necessary to elucidate the risk of gastrointestinal adverse events in different patient populations, particularly with respect to personalized treatment strategies for high-risk groups, including sex-specific analyses to address potential differences in nausea and vomiting susceptibility.

The findings of this study hold important implications for clinical practice. Although GLP-1RAs are widely utilized for glycemic control and weight management in T2DM, gastrointestinal adverse events remain a significant barrier to patient adherence. Clinicians should carefully consider individual patient characteristics, especially in those with preexisting gastrointestinal conditions, when selecting GLP-1RAs and adjusting dosages. Future research should also investigate strategies to minimize GLP-1RA-induced gastrointestinal adverse events, potentially through drug design innovations or combination therapies, to enhance patient tolerance and adherence.

## 5 Conclusion

This study highlights the varying risks of gastrointestinal adverse events associated with GLP-1 receptor agonists, with nausea being most common and reduced appetite least frequent. Short-acting GLP-1RAs carried a higher risk of nausea and vomiting, while long-acting agents were linked to increased diarrhea. Tirzepatide showed the highest overall risk. These findings emphasize the importance of individualized treatment approaches to optimize both efficacy and tolerability.

## Data Availability

The original contributions presented in the study are included in the article/Supplementary Material, further inquiries can be directed to the corresponding author.
