# Supplementary material for: Comparative gastrointestinal adverse effects of GLP-1 receptor agonists and multi-target analogs in type 2 diabetes: a Bayesian network meta-analysis
Source: Front Pharmacol. 2025 Sep 19;16:1613610. doi: 10.3389/fphar.2025.1613610 (PMC12491879; doi:10.3389/fphar.2025.1613610)
Supplement: Supplementary file 1 [file DataSheet1.docx]

1. ***PubMed search strategy***

*("GLP-1 Receptor Agonists"[MeSH Terms] OR "GLP-1 receptor agonists"[Title/Abstract] OR exenatide[Title/Abstract] OR liraglutide[Title/Abstract] OR dulaglutide[Title/Abstract] OR lixisenatide[Title/Abstract] OR benaglutide[Title/Abstract] OR loxenatide[Title/Abstract] OR semaglutide[Title/Abstract] OR tirzepatide[Title/Abstract]) AND ("Gastrointestinal Diseases"[MeSH Terms] OR gastrointestinal[Title/Abstract] OR nausea[Title/Abstract] OR vomiting[Title/Abstract] OR constipation[Title/Abstract] OR diarrhea[Title/Abstract] OR dyspepsia[Title/Abstract] OR "loss of appetite"[Title/Abstract]) AND (randomized[Title/Abstract] OR controlled[Title/Abstract] OR trial[Title/Abstract]) NOT ("type 1 diabetes"[Title/Abstract] OR pregnancy[Title/Abstract] OR breastfeeding[Title/Abstract])*

1. ***Embase search strategy***

*('glucagon like peptide 1 receptor agonist'/exp OR 'glp 1 receptor agonist'/exp OR exenatide:ti,ab OR liraglutide:ti,ab OR dulaglutide:ti,ab OR lixisenatide:ti,ab OR benaglutide:ti,ab OR loxenatide:ti,ab OR semaglutide:ti,ab OR tirzepatide:ti,ab) AND ('gastrointestinal disease'/exp OR gastrointestinal:ti,ab OR nausea:ti,ab OR vomiting:ti,ab OR constipation:ti,ab OR diarrhea:ti,ab OR dyspepsia:ti,ab OR 'loss of appetite':ti,ab) AND (randomized:ti,ab OR controlled:ti,ab OR trial:ti,ab OR 'clinical trial'/exp) NOT ('type 1 diabetes':ti,ab OR pregnancy:ti,ab OR breastfeeding:ti,ab)*

1. ***Cochrane Library search strategy****:*

*("GLP-1 receptor agonist" OR exenatide OR liraglutide OR dulaglutide OR lixisenatide OR benaglutide OR loxenatide OR semaglutide OR tirzepatide) AND (gastrointestinal OR nausea OR vomiting OR constipation OR diarrhea OR dyspepsia OR appetite) AND (randomized OR controlled OR trial) IN Trials*

1. ***ClinicalTrials.gov search strategy****:*

*GLP-1 receptor agonist OR exenatide OR liraglutide OR dulaglutide OR lixisenatide OR benaglutide OR loxenatide OR semaglutide OR tirzepatide AND gastrointestinal OR nausea OR vomiting OR constipation OR diarrhea OR dyspepsia OR loss of appetite AND randomized*
